# Which Strategies Reduce Breast Cancer Mortality Most? Collaborative Modeling of Optimal Screening, Treatment, and Obesity Prevention

**DOI:** 10.1002/cncr.28087

**Published:** 2013-04-26

**Authors:** Jeanne Mandelblatt, Nicolien van Ravesteyn, Clyde Schechter, Yaojen Chang, An-Tsun Huang, Aimee M Near, Harry de Koning, Ahmedin Jemal

**Affiliations:** 1 Department of Oncology, Georgetown University, Lombardi Comprehensive Cancer CenterWashington, DC; 2 Department of Public Health, Erasmus MCRotterdam, the Netherlands; 3 Departments of Family and Social Medicine and Epidemiology/Population Health, Albert Einstein School of MedicineBronx, New York; 4 American Cancer SocietyAtlanta, Georgia

**Keywords:** simulation, modeling, breast cancer, treatment, mammography, obesity

## Abstract

**BACKGROUND:**

US breast cancer mortality is declining, but thousands of women still die each year.

**METHODS:**

Two established simulation models examine 6 strategies that include increased screening and/or treatment or elimination of obesity versus continuation of current patterns. The models use common national data on incidence and obesity prevalence, competing causes of death, mammography characteristics, treatment effects, and survival/cure. Parameters are modified based on obesity (defined as BMI ≥ 30 kg/m^2^). Outcomes are presented for the year 2025 among women aged 25+ and include numbers of cases, deaths, mammograms and false-positives; age-adjusted incidence and mortality; breast cancer mortality reduction and deaths averted; and probability of dying of breast cancer.

**RESULTS:**

If current patterns continue, the models project that there would be about 50,100-57,400 (range across models) annual breast cancer deaths in 2025. If 90% of women were screened annually from ages 40 to 54 and biennially from ages 55 to 99 (or death), then 5100-6100 fewer deaths would occur versus current patterns, but incidence, mammograms, and false-positives would increase. If all women received the indicated systemic treatment (with no screening change), then 11,400-14,500 more deaths would be averted versus current patterns, but increased toxicity could occur. If 100% received screening plus indicated therapy, there would be 18,100-20,400 fewer deaths. Eliminating obesity yields 3300-5700 fewer breast cancer deaths versus continuation of current obesity levels.

**CONCLUSIONS:**

Maximal reductions in breast cancer deaths could be achieved through optimizing treatment use, followed by increasing screening use and obesity prevention. ***Cancer* 2013;119:2541–2548**. © *2013 American Cancer Society*.

## INTRODUCTION

Breast cancer mortality continues to decrease in the United States, largely because of improved treatment and screening,^1^ but it remains the most commonly diagnosed nonskin cancer and the second-leading female cause of cancer death, with about 40,000 dying each year.^2^ Reasons for the continuing burden of breast cancer are multifactorial and include the “graying of America,” high rates of obesity that affect incidence and complicate treatment, suboptimal access to screening and timely diagnostic follow-up, nonstandard and/or delayed treatment, limits in existing screening and therapeutic paradigms, socioeconomic factors that diminish survival, and unknown aspects of this disease.^3^–^9^ Evaluating the impact of these factors on breast cancer mortality across the population separately and jointly is not feasible through empirical research.

Modeling can be used as a population laboratory to estimate the impact of changing a number of these contributing factors alone or in combination.^10^–^12^ In this article, we use 2 well-established models^1^,^11^–^15^ to evaluate combinations of screening and treatment strategies^16^ or elimination of obesity to decrease breast cancer deaths beyond what would be expected if current patterns persist.

## MATERIALS AND METHODS

The 2 models (MISCAN-Fadia and SPECTRUM) were developed independently within the Cancer Intervention and Surveillance Modeling Network^11^,^12^,^17^ and were exempt from institutional review board approval. The models estimate the impact of applying 6 strategies in the US female population from 2012 to 2025 versus maintaining current patterns: 1) 90% of women screen annually from ages 40 to 54 and biennially from ages 55 to 99 (or death) and the remaining 10% do not screen at all; women receive treatment based on current patterns; 2) current screening, but 100% receive treatment indicated by age, stage, and ER/HER2 status^18^; 3) 90% screening and 100% receipt of indicated treatment; 4) 100% screening and current patterns of treatment; 5) 100% screening and 100% indicated treatment; and 6) eliminate obesity but maintain current screening and treatment. Although we will never achieve 100% compliance or eliminate obesity, these strategies demonstrate upper bounds of possible known approaches. We examined a hybrid strategy of more frequent screening intervals at younger ages than at older ages because there are shorter age-dependent sojourn times before versus after menopause. We did not impose an upper age limit to provide an estimate of the impact of screening and treatment over the entire life course.

### Model Overview

Both models begin with estimates of incidence and mortality trends without screening and systemic treatment and then overlay screening use and improvements in survival associated with systemic therapy.^19^ We overlay actual dissemination of screening and systemic treatments as our “base case,” carrying these rates forward into the future. We superimpose the 6 strategies beginning in 2012 through 2025. Women are followed until death, even if that date is after 2025.

Breast cancer is depicted as having a preclinical screening-detectable period and a clinical detection point. On the basis of mammography sensitivity (or thresholds of detection), screening identifies disease in the preclinical period and results in the identification of earlier-stage or smaller tumors than occurs via clinical detection. In MISCAN-Fadia, treatment results in cure for some women, and in SPECTRUM it results in reductions in the hazards of death. Obesity (body mass index [BMI] ≥ 30 kg/m^2^) affects outcomes based on its age- and cohort-specific prevalence^20^–^23^ through its impact on multiple model parameters.^15^ Current obesity prevalence is projected forward.^24^

### Model Parameters

Both models use a common set of age-specific variables along with model-specific inputs to represent disease history (eg, incidence, stage shifts, or tumor growth).^1^,^10^–^12^ For instance, based on the varying model structures, SPECTRUM uses an age–period–cohort model^25^ to represent incidence from 1975 to 2000 without screening, whereas MISCAN-Fadia only uses it to estimate tumor onset. Consequently, the models have slightly different incidence rates beginning in 1975, but comparable results for trends over time.^11^,^12^,^14^ Both extrapolate age-specific incidence rates forward based on rates in 2000. Obesity increases the risk for breast cancer in postmenopausal women (relative risk [RR], 1.25) but decreases the risk in premenopausal women (RR, 0.60).^7^,^8^

The current dissemination of mammography is depicted based on the age of receipt of the first mammography and the interval between subsequent mammograms using data from the National Health Interview Survey and the Breast Cancer Surveillance Consortium (BCSC), respectively; current rates are carried forward to 2025 for each cohort and age group.^26^,^27^ Because mammography use does not vary by BMI except at extremes values,^28^ we assume that obesity has no effect on mammography use. We use age- and BMI-specific mammography sensitivity and specificity observed in the BCSC (unpublished data) to develop model inputs^15^,^29^ and with other data^11^ to define thresholds of detection. The impact of digital mammography was evaluated in sensitivity analyses.

The American Joint Committee on Cancer stage distribution in the absence of screening is estimated from Surveillance, Epidemiology, and End Results (SEER) data in 1975-1979.^30^ After 1979 we phase in distributions among clinically detected women using BCSC data. Stage distributions among screened women are estimated using unpublished BCSC data from 1996 to 2007. Because obesity is associated with more advanced tumors at diagnosis,^31^,^32^ we use BCSC data on stage by BMI and age groups for unscreened and screened women.

The joint distribution of ER and HER2 status by age, year, and stage is estimated from women diagnosed from 1997 to 2005.^14^,^33^ Because obesity affects the rate of ER-positive tumors differentially by menopausal status,^34^ we apply RRs of 0.86 and 1.78 to the probability of ER-positive cancer among obese pre- and postmenopausal women, respectively. We assume that obesity has no impact on HER2 status.

Dissemination of systemic chemo- and hormonal therapy from 1975 to 2000 was estimated using NCI Patterns of Care data by age, year, stage, and ER status^35^,^36^ and updated through 2010 (including trastuzumab for HER2-positive cases) using unpublished data from the National Comprehensive Cancer Network Outcomes Database; these data are carried forward. Strategies that include 100% indicated treatment assume: 1) ER-positive invasive cases receive chemotherapy and hormonal treatment based on age and year (tamoxifen between 1980 and 1999, tamoxifen <50 years, and anastrozole if ≥50 years from 2000 to 2010), and DCIS cases only receive hormonal therapy; 2) ER-negative invasive cases receive chemotherapy; and 3) HER2-positive tumors diagnosed in 2005 or later receive trastuzumab.^18^,^35^,^36^ We assume that treatment patterns do not vary by obesity. Treatment effectiveness is based on a synthesis of clinical trials.^37^–^40^ Chemotherapy effectiveness is reduced in about 30% of obese ER-negative women based on dose reductions.^3^,^5^,^6^ We assume obesity has no impact on hormonal or trastuzumab effectiveness.^8^ SEER data from 1975 to 1979 are used to estimate breast cancer survival before screening and adjuvant treatment were available.^30^ Nonbreast cancer mortality is calculated by subtracting breast cancer from all-cause mortality.^41^–^43^ The impact of obesity was incorporated using NHANES-mortality linked data.^21^

### Benefits and Burden

The models project the probability of breast cancer death and age-adjusted mortality rates from age 25 years to death based on continuing current patterns of screening and treatment and each of the 6 alternative strategies in 2025. The number of breast cancer deaths averted is calculated by applying model projections of age-specific mortality rates to the age-specific US population projections for 2025.^44^ False-positive mammograms are a proxy for burden and are defined as the number of mammograms read as needing further follow-up in women without cancer. Results are presented in absolute terms and incremental numbers compared with current patterns.

## RESULTS

**Figure 1:**
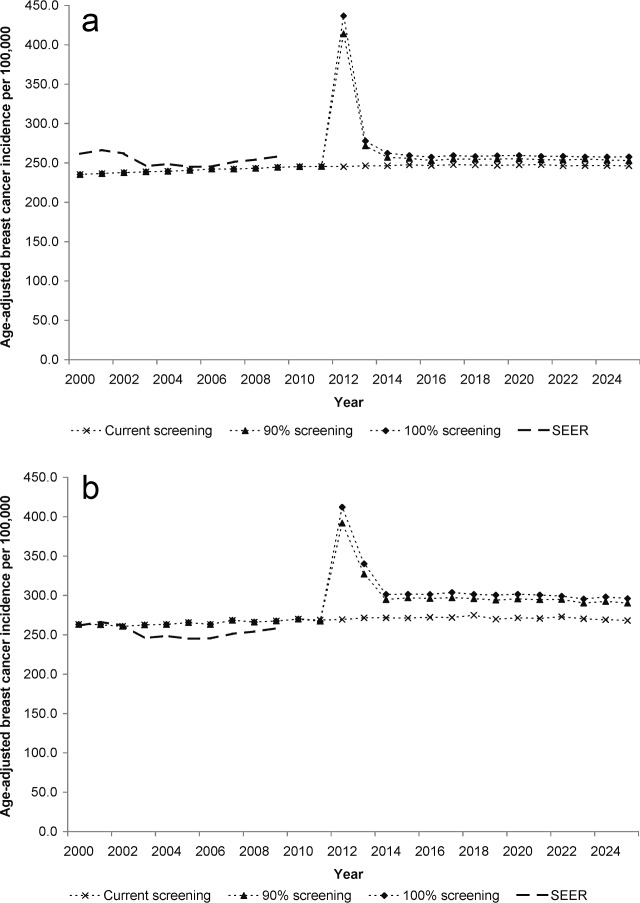
Age-adjusted breast cancer incidence rates from 2000 to 2025 predicted by the models for alternative screening strategies (strategies that include treatment are not included since they do not affect incidence) versus those reported to SEER (breast cancer incidence reported to SEER from 2000 to 2009) for women 25 years and older. (a) SPECTRUM. (b) MISCAN-Fadia.

**Figure 2:**
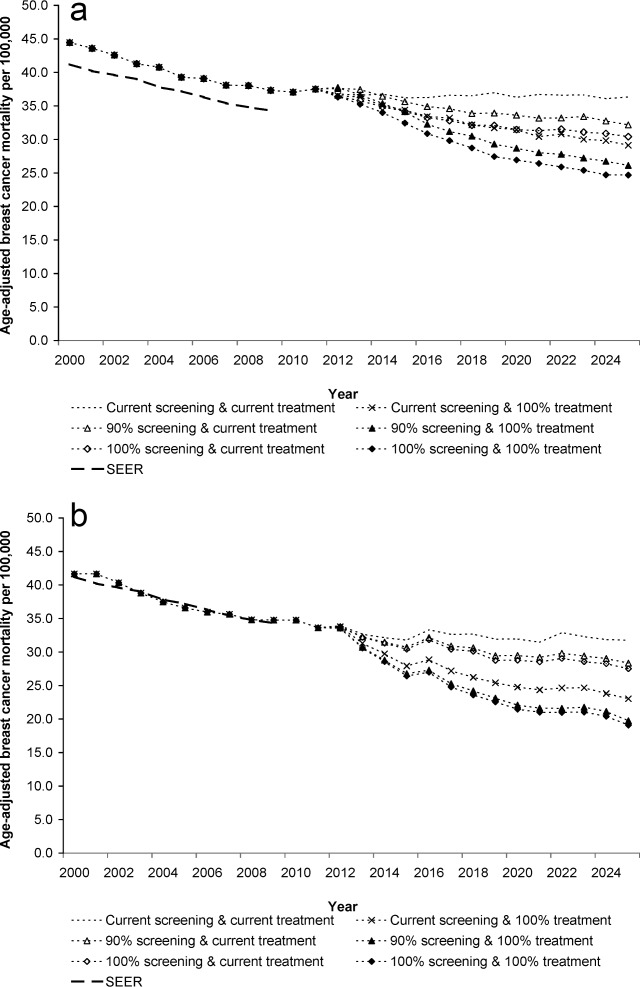
Predicted age-adjusted breast cancer mortality from 2000 to 2025 by alternative screening and treatment strategies versus that reported to SEER (breast cancer mortality reported in SEER from 2000 to 2009) for women 25 years and older. (a) SPECTRUM. (b) MISCAN-Fadia.

Observed incidence and mortality rates (Figs. 1 and 2) and stage distributions (not shown) from 2000 to 2009 were accurately reproduced by both models. If current patterns continue to 2025, the models project that breast cancer mortality rates would be 31.8-36.3 (range across the models) per 100,000 women, or 50,000-57,400 breast cancer deaths in women aged 25 or older (Table 1).

**TABLE 1:** Predicted Absolute Outcomes in 2025 by Model and Alternative Screening and Treatment Strategies Versus Continuation of Current Patterns for Women 25 Years and Older

	No. of Mammograms/1000		False-Positives/1000		Age-Adjusted Mortality Rate/100,000		Number of Breast Cancer Deaths^d^		Probability of Dying of Breast Cancer^e^	
					
Strategy	SPECTRUM	MISCAN-Fadia	SPECTRUM	MISCAN-Fadia	SPECTRUM	MISCAN-Fadia	SPECTRUM	MISCAN-Fadia	SPECTRUM	MISCAN-Fadia
Current screening and Rx patterns^a^	261.6	278.1	30.9	28.4	36.3	31.8	57,400	50,100	2.9%	2.9%
Current screening and 100% Rx^b^	261.4	277.9	30.9	28.4	29.1	23.0	46,000	35,600	2.3%	2.1%
90% screening and current Rx^a^,^c^	417.9	419.7	51.8	48.2	32.2	28.4	51,300	45,000	2.5%	2.6%
90% screening and 100% Rx^b^,^c^	417.7	419.4	51.9	48.1	26.1	19.7	41,700	30,700	2.1%	1.8%
100% screening and current Rx^a^,^c^	464.2	465.9	57.6	53.5	30.4	27.5	48,400	43,600	2.4%	2.5%
100% screening and 100% Rx^b^,^c^	463.9	465.6	57.5	53.5	24.7	19.1	39,300	29,700	2.0%	1.7%

Abbreviations: Rx, treatment.

a Current refers to screening and/or treatment as actually disseminated in the US population.

b All women receive indicated treatment based on age, stage, and ER/HER2 status.

c Ninety percent or 100% schedules are annual screening from ages 40 to 54 and biennially from ages 55 to 99 (or death). In the 90% strategy, the remaining 10% are assumed to not have any screening.

d Rounded to the nearest hundred.

e Calculated using Probability of Developing or Dying of Cancer Software, Version 6.6.1; Surveillance Research Program, Statistical Methodology and Applications Branch, National Cancer Institute, 2012; http://surveillance.cancer.gov/devcan.

### Increased Screening or Indicated Treatment Versus Continuation of Current Patterns

If screening rates increase in 2012 from current patterns to 90% of women screened annually from age 40 to 54 and then biennially from age 55 onward (with no change in treatment patterns), incidence rates would have a transient increase at the start, followed by a leveling off at a higher rate than seen presently (Fig. 1). The higher incidence would be accompanied by mortality reductions of 10.7%-11.5% in 2025 compared with continuing current screening, or about 5100- 6100 deaths averted among women ≥25 years (Table 2 and Fig. 2). These benefits would require more than 140 additional mammograms per 1000 women in 2025, including about 20 more false-positive tests per 1000 women than would occur if current patterns continue (Table 2).

**TABLE 2:** Predicted Incremental Outcomes in 2025 by Model and Alternative Screening and Treatment Scenario Versus Continuation of Current Patterns for Women 25 Years and Older^a^

	Mammograms/1000		False-Positives/1000		Percent Mortality Reduction		Breast Cancer Deaths Averted^d^	
				
Strategy (Each Compared Incrementally To Current)	SPECTRUM	MISCAN-Fadia	SPECTRUM	MISCAN-Fadia	SPECTRUM	MISCAN-Fadia	SPECTRUM	MISCAN-Fadia
Current screening and Rx patterns^a^	—	—	—	—	—	—	—	—
Current screening and 100% Rx^b^	NA	NA	NA	NA	19.8	27.5	11,400	14,500
90% screening and current Rx^a^,^c^	156.3	141.6	20.9	19.8	11.5	10.7	6100	5100
90% screening and 100% Rx^b^,^c^	156.1	141.3	21.0	19.7	28.1	37.9	15,700	19,400
100% screening and current Rx^a^,^c^	202.6	187.8	26.7	25.1	16.3	13.4	8900	6500
100% screening and 100% Rx^b^,^c^	202.3	187.5	26.6	25.1	32.1	39.9	18,100	20,400

Abbreviations: NA, not applicable because no change in screening.

a Current refers to screening and/or treatment as actually disseminated in the US population.

b All women receive indicated treatment based on age, stage, and ER/HER2 status.

c Ninety percent or 100% schedules are annual screening from ages 40 to 54 and biennially from ages 55 to 99 (or death). In the 90% strategy, the remaining 10% are assumed to not have any screening.

d Rounded to the nearest hundred.

If 100% of women are screened, incidence increases further (Fig. 1), but mortality could be reduced (Fig. 2) by 13.4%-16.3% in 2025 versus continuation of current screening use. This translates into almost 6500-8900 more deaths averted than continuation of current patterns, but with an even greater increase in mammograms and false-positives (Table 2). However, if screening continues at current levels, but all women receive indicated therapy, then mortality rates could be decreased by 19.8%-27.5% versus continuation of current treatment patterns, and 11,400-14,500 deaths could be avoided (Table 2 and Fig. 2).

### One Hundred Percent Screening and 100% Indicated Treatment Versus Continuation of Current Patterns

Optimizing screening and treatment could reduce mortality by less than the sum of each approach because they interact (eg, the better treatment is, the less screening contributes to mortality reduction). Thus, the maximum aggregate reductions that could be achieved under optimal conditions are about 18,100- 20,400 more deaths averted in 2025 versus maintaining current patterns (Table 2 and Fig. 2). This corresponds to reducing a woman's lifetime probability of dying of breast cancer after age 25 years from 2.9% to 1.7%-2.0% (range across models); see Table 1. However, even under these idealized circumstances, there would still be 29,700-39,300 breast cancer deaths.

### Elimination of Obesity

Obesity increases the incidence of breast cancer; 5.4%-5.6% of cases expected to occur in 2025 would be attributable to obesity if current rates are maintained (Table 3). If we could eradicate obesity, there could be about 3300-5700 fewer breast cancer deaths in 2025 in women ≥25 years.

**TABLE 3:** Projected Impact of Obesity on Breast Cancer Outcomes for US Women 25 and Older in 2025 Assuming Current Patterns of Care Are Maintained

	Obese		Nonobese		All Women	
			
Incidence	SPECTRUM	MISCAN-Fadia	SPECTRUM	MISCAN-Fadia	SPECTRUM	MISCAN-Fadia
Age-adjusted incidence rate per 100,000	270.7	287.0	241.9	252.5	251.5	263.4
No. of breast cancer cases (invasive and in situ)^a^	157,200	167,100	228,700	245,600	385,900	412,700
Attributable fraction of breast cancer cases due to obesity^b^	—	—	—	—	5.4%	5.6%
No. of cases that could be avoided if obesity were eliminated^a^	—	—	—	—	20,700	23,000
**Mortality**						
Age-adjusted mortality rate per 100,000	40.3	35.2	30.8	29.8	33.8	31.6
Percent mortality reduction^c^	—	—	—	—	9.1%	6.4%
No. of breast cancer deaths^a^	23,100	20,100	32,700	30,000	55,900	50,100
Attributable fraction of breast cancer deaths due to obesity^d^	—	—	—	—	10.2%	6.6%
No. of deaths that could be averted if obesity were eliminated^a^	—	—	—	—	5,700	3,300

a Rounded to the nearest hundred.

b Attributable fraction of incident cases based on formula p^*^(i_O − i_N)/(p^*^i_O + (1 − p)^*^i_N), where p is prevalence of obesity, i_O is incidence in obese, and i_N is incidence in nonobese.

c Percent mortality reduction is calculated as the difference in the age-adjusted breast cancer mortality in 2025 between the current pattern and the nonobese scenario divided by the age-adjusted mortality in the current pattern scenario.

d Attributable fraction of deaths based on formula p^*^(m_O − m_N)/(p^*^m_O + (1 −p)^*^m_N), where p is prevalence of obesity, m_O is mortality in obese, and m_N is mortality in nonobese.

### Sensitivity Analysis

Improving test sensitivity or switching to digital mammography does not change the results substantially (not shown) because most lesions are slow growing and if missed on 1 exam are detected on the subsequent screen without much impact on mortality.

## DISCUSSION

Maximal reductions in US deaths from breast cancer might be achieved through ensuring that all women have clinically indicated systemic therapy, followed by increasing screening, then obesity prevention after age 50, although greater screening could exacerbate false-positives and increase incidence. Even if optimal deployment of these currently available breast cancer control strategies were achievable, the number of projected future breast cancer deaths remains high.

Optimizing use of currently available systemic therapies results in nearly double the number of deaths averted than with enhancing screening levels compared with current patterns. But greater uptake of systemic therapy could lead to more therapy-related toxicity, and there are barriers to use at the system, provider, and patient level. For instance, suboptimal compliance with the full course of hormonal therapy has been noted in other research, so that modeled mortality reductions may not be realized.^45^,^46^

In past research, we examined differences between lifetime screening annually or biennially starting at age 40 or 50.^13^ In the current study, we have extended those results by examining a hybrid approach of screening annually starting at age 40 and changing to a biennial schedule at age 55 and including different rates of use. The results suggest that the majority of added benefit of increased screening use is from increasing screening levels to 90% of women using regular screening compared with current patterns, even though 100% compliance could avoid additional deaths. However, recommendations for and compliance with 90%-100% regular screening at all ages will be difficult to acheive,^47^,^48^ so that fewer deaths will be averted than projected by the models. Additional screening also imposes a burden of added false-positive results and increased incidence (and overdiagnosis).^13^,^49^,^50^

Greater program efficiencies might be achieved by using a community-based approach in populations in which screening and treatment services are suboptimal, as well as “personalized” risk-based approaches to target screening and treatment. The latter approach could result in more intensive screening of women with the highest risk of developing disease and deployment of therapies by women most likely to benefit and decreased use by women unlikely to benefit, minimizing harm and toxicity. However, to date, there is only a limited empiric database to support personalized approaches.^51^,^52^ Future modeling should consider the impact of individual risk-based cancer control strategies as well as targeting geographic areas and communities with the highest burden of cancer and the least resources.

The models estimate that obesity, which presently occurs in about one third of the female population older than age 50,^20^ accounts for only a modest number of breast cancer cases and deaths. Moreover, these estimates are an upper bound of what is achievable with intensive campaigns to lower obesity rates. As more data become available, it will be interesting to reexamine how strategies to reduce obesity will affect breast cancer outcomes via influences on the immune and metabolic systems that are implicated in breast cancer risk or probability of recurrence.^53^

Overall, the collaboration of 2 groups with different modeling approaches and structures to estimate the same end points by using common data provides a reasonable range of expected results. Despite these strengths and our consistent results, our study has limitations. We do not capture decrements in quality of life associated with false-positive results, living with earlier knowledge of a cancer diagnosis and possible side effects of treatment, or overdiagnosis.^50^,^54^ We extrapolate current data forward, and patterns may not continue as projected. We include mammography resources and do not include resources associated with increased use of therapy. The models also do not consider other primary prevention approaches beyond obesity reduction (eg, tamoxifen use by high-risk women) or improvements in multimodality local therapy over time.

In summary, our results suggest that substantial improvements in US breast cancer control can be made by ensuring that all women receive indicated systemic therapy, use regular screening, and avoid obesity after age 50. Multiple leverage points will be required to realize these improvements, but increasing the use of indicated systemic therapy is a necessary component of strategies for women diagnosed with breast cancer. Combinations of other approaches and new paradigms, guided by evidence from modeling, novel trials, and new scientific discovery, will be needed for further reductions in the future burden of breast cancer.
